# In vitro and clinical investigations to determine the drug-drug interaction potential of entrectinib, a small molecule inhibitor of neurotrophic tyrosine receptor kinase (*NTRK*)

**DOI:** 10.1007/s10637-021-01156-9

**Published:** 2021-08-21

**Authors:** Georgina Meneses-Lorente, Stephen Fowler, Elena Guerini, Karey Kowalski, Edna Chow-Maneval, Li Yu, Francois Mercier, Mohammed Ullah, Kenichi Umehara, Andreas Brink, Vincent Buchheit, Elke Zwanziger, Alex Phipps, Nassim Djebli

**Affiliations:** 1 Roche Products Ltd, Welwyn Garden City, UK; 2 Roche Innovation Center Basel, F. Hoffmann-La Roche Ltd, Basel, Switzerland; 3 Ignyta, Inc, San Diego, CA USA; 4 Roche Innovation Center, Little Falls, NJ USA

**Keywords:** Entrectinib, TRK/ROS1/ALK, Drug-drug interactions, CYP3A4, P-glycoprotein

## Abstract

*Background* Entrectinib is a CNS-active, potent inhibitor of tyrosine receptor kinases A/B/C, ROS1 and anaplastic lymphoma kinase approved for use in patients with solid tumors. We describe the in vitro and clinical studies investigating potential entrectinib drug-drug interactions. *Methods* In vitro studies with human biomaterials assessed the enzymes involved in entrectinib metabolism, and whether entrectinib modulates the activity of the major cytochrome P450 (CYP) enzymes or drug transporter P-glycoprotein. Clinical studies investigated the effect of a strong CYP3A4 inhibitor (itraconazole) and inducer (rifampin) on single-dose entrectinib pharmacokinetics. The effect of entrectinib on sensitive probe substrates for CYP3A4 (midazolam) and P-glycoprotein (digoxin) were also investigated. *Results* Entrectinib is primarily metabolized by CYP3A4. In vitro, entrectinib is a CYP3A4/5 inhibitor (IC_50_ 2 μM) and a weak CYP3A4 inducer. Entrectinib inhibited P-glycoprotein (IC_50_ 1.33 μM) but is a poor substrate. In healthy subjects, itraconazole increased entrectinib C_max_ and AUC by 73% and 504%, respectively, and rifampin decreased entrectinib C_max_ and AUC by 56% and 77%, respectively. Single dose entrectinib did not affect midazolam AUC, although C_max_ decreased by 34%. Multiple dose entrectinib increased midazolam AUC by 50% and decreased C_max_ by 21%. Single dose entrectinib increased digoxin AUC and C_max_ by 18% and 28%, respectively, but did not affect digoxin renal clearance. *Conclusions* Entrectinib is a CYP3A4 substrate and is sensitive to the effects of coadministered moderate/strong CYP3A4 inhibitors and strong inducers, and requires dose adjustment. Entrectinib is a weak inhibitor of CYP3A4 and P-glycoprotein and no dose adjustments are required with CYP3A4/P- glycoprotein substrates.

*Registration Number (Study 2)* NCT03330990 (first posted online November 6, 2017) As studies 1 and 3 are phase 1 trials in healthy subjects, they are not required to be registered.

**Supplementary Information:**

The online version contains supplementary material available at 10.1007/s10637-021-01156-9.

## Introduction

Entrectinib (also known as RXDX-101 and Rozlytrek®) is a CNS-active, potent and selective inhibitor of tyrosine receptor kinases (TRK) A/B/C, ROS1 and anaplastic lymphoma kinase (ALK), which are encoded by the genes neurotrophic tyrosine receptor kinase (NTRK) 1/2/3, ROS1, and ALK, respectively. These kinases are overexpressed or dysregulated in cancer with constitutive activity, making the growth of the cancer cells dependent on the abnormal kinases [1, 2]. Molecular alterations in kinases are found in many types of cancer and therefore represent attractive targets for anticancer therapy [3]. Entrectinib has been shown to have antitumor activity in advanced and/or metastatic solid tumors [4–6], and has recently been approved in the several regions (including the US, EU, Japan and Canada) for the treatment of adult and/or pediatric patients with tumors that harbor NTRK1/2/3 or ROS1 gene rearrangements. The recommended dosage in adults is 600 mg orally once daily with or without food.

In a mass balance study, entrectinib has been shown to be mainly eliminated through hepatic clearance, with negligible renal clearance (< 1% of the dose is excreted unchanged in urine) [7]. There are two major circulating metabolites of entrectinib in humans, M5 and M11, contributing 12% and 19%, respectively, of total circulating radioactivity. M5 is a product of *N-*demethylation and M11 is formed by *N*-glucuronidation. M5 is pharmacologically active and therefore its pharmacokinetics have been assessed during entrectinib development. M11 is not pharmacologically active and as a glucuronide was not considered MIST-relevant [8].

As entrectinib is used to treat cancer patients, it is expected to be coadministered with many other drugs including known drug metabolizing enzyme inhibitors such as antivirals (eg, ritonavir), macrolide antibiotics (eg, telithromycin), antifungals (eg, itraconazole) and inducers such as rifampin, carbamazepine and phenytoin. It is therefore important to understand the potential for drug-drug interactions (DDIs) both with respect to entrectinib as a victim and as a perpetrator. In vitro and clinical studies were conducted to assess the DDI potential of entrectinib. In vitro studies using human hepatocytes, human liver microsomes and cDNA-expressed human cytochrome P450 (CYP) enzymes, investigated which enzymes are primarily involved in the metabolism of entrectinib and in the formation of M5. In vitro studies also assessed whether entrectinib and M5 are substrates for drug transporters. The results of these studies were used to guide targeted clinical assessment of potential DDIs with drugs known to be strong inhibitors or inducers of the relevant enzymes and/or transporters to assess the magnitude of any changes in entrectinib exposure. Further in vitro studies using human hepatocytes, human liver microsomes, human P-glycoprotein (P-gp) expressing cell lines, and Caco-2 and MDCK11-MDR1 cells, were designed to investigate the potential of entrectinib and M5, to modulate the activity of CYP enzymes and P-gp. The results of these studies were used to guide further clinical studies to assess whether coadministration of entrectinib with substrates of these enzymes/transporters lead to changes in the substrate’s exposure.

Here we report a summary of the in vitro studies conducted with entrectinib and M5, and the results of 3 clinical DDI studies conducted in either cancer patients or healthy subjects. The first clinical study described here assessed the effect of a strong CYP3A4 inhibitor (itraconazole) and a strong CYP3A4 inducer (rifampin) on the pharmacokinetics of entrectinib and M5. The second clinical study assessed the effect of entrectinib on the pharmacokinetics of midazolam, a sensitive CYP3A4 substrate. The third clinical study investigated the effect of entrectinib on the pharmacokinetics of digoxin, a sensitive substrate of P-glycoprotein (P-gp). The results of these studies were used to guide dosing recommendations for entrectinib.

## Methods

### In vitro studies

The metabolic profile of entrectinib has been investigated in vitro in studies using [14] Centrectinib in both human liver microsomes and human hepatocytes. In addition, the metabolism of entrectinib was investigated using 12 cDNA-expressed human CYP enzymes: CYPs 1A1, 1A2, 2A6, 2B6, 2C8, 2C9, 2C18, 2C19, 2D6, 2E1, 3A4 and 3A5. The effect of CYP-selective chemical inhibitors on the metabolism of entrectinib by pooled human liver microsomes was also investigated. The selective inhibitors used were α-naphthoflavone (CYP1A2), ticlopidine (CYP2B6), montelukast (CYP2C8), sulfaphenazole (CYP2C9), benzylphenobarbital (CYP2C19), quinidine (CYP2D6) and ketoconazole (CYP3A).

The in vitro inhibition of human CYPs 1A2, 2B6, 2C8, 2C9, 2C19, 2D6 and 3A by entrectinib and M5 was investigated in human liver microsomes. In vitro induction of CYP mRNA (CYPs 1A2, 2C8, 2C9 and 3A4) or enzyme activity (CYPs 1A2, 2C19 and 3A4) was assessed in human hepatocytes.

The potential of entrectinib and M5 to be substrates of P-gp was assessed in studies in Caco-2 cells and MDCKII-MDR1 cells. In addition, the potential for entrectinib and M5 to inhibit P-gp was also assessed.

Outlines of the in vitro studies describing key reagents, timepoints and conditions are included in the supplementary material.

### Clinical studies

#### Clinical study design

In all 3 studies, subjects provided written informed consent and underwent screening procedures to confirm eligibility for each study within 3 to 4 weeks prior to receiving any study medication.

Study 1 was an open-label, fixed-sequence, 2-cohort, single-center study in healthy adult subjects to evaluate the effect of a strong CYP3A4 inhibitor (itraconazole) and a strong CYP3A4 inducer (rifampin) on the pharmacokinetics of entrectinib and M5. In Cohort 1, subjects received a single dose of 100 mg entrectinib on Day 1 (Period 1). This dose was chosen as it was expected that when it was administered with a strong CYP3A4 inhibitor entrectinib exposure would not exceed the exposure seen with 600 mg entrectinib alone. After a 9-day washout, subjects received 200 mg once daily (QD) itraconazole on Days 10 through 19 and a single dose of 100 mg entrectinib 1 h after itraconazole on Day 14 (Period 2). Entrectinib was administered 1 h later than itraconazole in an attempt to match the peak concentrations for both compounds to maximize the potential for interactions. On Days 1 and 14, subjects were fasted for 10 h prior to, and for 4 h after entrectinib dosing. Subjects were resident in the study center from Day -1 through Day 3, and from Day 13 through Day 16. All doses of study medication were administered at the study center. Blood samples for entrectinib and M5 plasma concentrations were collected at intervals from prior to entrectinib dosing through to 144 h after entrectinib dosing on Days 1 and 14.

In Cohort 2, subjects received a single dose of 600 mg entrectinib on Day 1 (Period 1). After a 9-day washout, subjects received 600 mg QD rifampin on Days 10 through 25 (Period 2) and a single dose of 600 mg entrectinib concurrently with rifampin on Day 21. On Days 1 and 21, subjects were fasted for 10 h prior to, and for 4 h after entrectinib dosing. Subjects were resident in the study center from Day -1 through Day 3, and from Day 20 through Day 23. All doses of study medication were administered at the study center. Blood samples for entrectinib and M5 plasma concentrations were collected at intervals from prior to entrectinib dosing through to 120 h after entrectinib dosing on Days 1 and 21.

Study 2 (NCT03330990) was an open-label, fixed-sequence, 3-center study in subjects with advanced solid tumors to evaluate the effect of multiple doses of entrectinib on the single-dose pharmacokinetics of midazolam and its active metabolite 1’-hydroxymidazolam. Subjects received 2 mg midazolam (midazolam hydrochloride syrup) orally on Days 1, 8 and 21 under fasted conditions. Entrectinib (600 mg/day orally) dosing began on Day 8 and continued through Day 22. Entrectinib was self-administered on Days 10 through 20. On Days 8 and 21, midazolam was administered 1 h after entrectinib. On Days 1, 8 and 21, blood samples for midazolam and 1’-hydroxymidazolam plasma concentrations were collected from 1 h prior to midazolam dose, and at intervals up to 34 h post-midazolam dose. Following completion of this part of the study, subjects could continue in an expanded access portion of the study which is not discussed here.

Study 3 was an open-label, fixed-sequence, single-center study in healthy adult subjects to evaluate the effect of entrectinib on the pharmacokinetics of oral digoxin. Subjects received a single dose of 0.5 mg digoxin on Day 1 (Period 1) under fasted conditions. After a 10-day washout, subjects received a single oral dose of 600 mg entrectinib, 1 h prior to administration of 0.5 mg digoxin on Day 11, under fasting conditions (Period 2). Subjects were resident in the study center from Day -1 through Day 4 and from Day 10 though Day 14. Blood samples for digoxin plasma concentrations were collected at intervals from prior to digoxin dosing through to 144 h after digoxin dosing on Days 1 and 11. Urine samples for digoxin concentrations were collected from prior to digoxin dosing and up to 72 h after digoxin dosing on Days 1 and 11.

#### Subjects

Studies 1 and 3 included healthy adult subjects (males only in Study 3). Key exclusion criteria included restrictions of any prescription drugs for at least 14 days (or 5 half-lives) prior to Day 1, any over-the-counter medication within 7 days of Day 1 (unless agreed by the Principal Investigator and Sponsor as not clinically relevant), and any investigational drug in any clinical trial within 30 days (or 5 half-lives) prior to Day 1. Use of drugs with enzyme-inducing properties or strong CYP3A4 inhibitors within 4 weeks prior to Day 1 were also excluded.

Study 2 included adult male and female subjects who had a histologically or cytologically confirmed diagnosis of advanced or metastatic solid tumors not responsive to standard therapies or for which there was not effective therapy. Subjects were initially required, and after an amendment, subsequently preferred to have tumors harboring NTRK1/2/3, ROS1, or ALK molecular alterations. Prior cancer therapy was allowed, but had to have been completed within prespecified time-limits prior to the start of midazolam dosing. Subjects had to have an ECOG performance status score of ≤ 1, and have adequate hematologic, liver and renal function. Use of strong CYP3A inhibitors or inducers was not allowed within 14 days prior to the start of midazolam dosing.

#### Pharmacokinetic assessments

Entrectinib and M5 plasma concentrations were measured using a validated liquid chromatography-tandem mass spectrometry (LC–MS/MS) method with a lower limit of quantification (LLOQ) of 2.00 ng/mL for both analytes. Midazolam and 1’-hydroxymidazolam plasma concentrations were measured using a validated LC–MS/MS method with an LLOQ of 0.100 ng/mL for both analytes. Digoxin plasma and urine concentrations were measured using a validated LC–MS/MS method with an LLOQ of 0.100 ng/mL (plasma) and 0.200 ng/mL (urine), respectively.

PK parameters were determined using noncompartmental analysis (Phoenix WinNonlin software, Certara, NJ, USA). PK parameters included maximum plasma concentration (C_max_), time to C_max_ (T_max_), area under the curve (AUC) from time zero to 24 h post dose (AUC_0-24_), AUC extrapolated to infinity (AUC_inf_), terminal half-life (t_1/2_), where appropriate. The molar AUC_inf_ and C_max_ ratios of 1’-hydroxymidazolam to midazolam were also calculated in Study 2. The percent of the dose of digoxin recovered unchanged in urine (f_e_) and renal clearance (CL_R_) were calculated from urine data in Study 3.

#### Statistical assessments and sample size

No formal calculations on sample size were made for any of the studies. Ten subjects in each cohort of Study 1, and in total in Study 3 were deemed sufficient based on similar phase 1 studies. In Study 2, up to 15 subjects were to be enrolled to provide a final dataset of at least 8 evaluable subjects which was considered typical for studies of this nature.

For all studies, a mixed effects model with fixed effect for treatment and a random subject effect were used to analyze the logarithms of AUC_inf_, and C_max_. For each treatment comparison, a point estimate and 90% confidence interval (CI) were provided for the geometric mean ratio. Lack of interaction was concluded if the 90% CI for the ratio was fully contained within 80% to 125% for each parameter.

## Results

### In vitro metabolism of entrectinib

Moderate turnover of entrectinib was observed in human hepatocytes, with 29% of the drug metabolized after 120 min of incubation. Similarly, 47% of parent remained after 60 min of incubation with pooled human liver microsomes supplemented with NADPH. The main metabolite formed in both profiling studies was M5 (*N-*demethylation at the piperazine). M5 accounted for 12% of total drug-related material (representing 41% of entrectinib metabolism) in human hepatocytes, and for 28% of the [14] Centrectinib-derived radioactivity in human liver microsomes. All other metabolites together accounted for less than 20% of metabolism in hepatocytes and less than 10% of the radioactivity in microsomes. Studies using long-term micropatterned co-cultured human hepatocytes suggested that M5 exhibited an approximately twofold lower intrinsic clearance than entrectinib.

Studies with CYP-selective inhibitors on the metabolism of entrectinib demonstrated a strong effect of ketoconazole (approximately 50–80% inhibition depending on the metabolite formed), indicating an important role for CYP3A enzymes in the oxidative metabolism of entrectinib (especially demethylation to M5). Other enzymes may also contribute to some extent, but no contributions exceeding 25% were observed for any of the metabolism pathways in vitro (Table 1). The CYP reaction phenotyping studies with cDNA-expressed human CYP enzymes, also suggested that entrectinib metabolites were formed by multiple CYPs but that CYP3A4 was substantially more active than the other enzyme preparations. CYP3A4 was also shown to catalyze both the generation and further metabolism of M5 (Table S1).

**Table 1:** Effect of CYP-Selective Inhibitors on the Metabolism of 1µM Entrectinib by Pooled Human Liver Microsomes

Test Inhibitor	Enzyme Target	% Inhibition of Entrectinib Metabolism to M5	Effect on Formation of other Entrectinib Metabolites
α-Naphthoflavone (0.5 µM)	CYP1A2	< 20	-
Ticlopidine (50 µM)	CYP2B6	< 20	-
Montelukast (3 µM)	CYP2C8	< 20	+
Sulfaphenazole (10 µM)	CYP2C9	< 20	-
Benzylphenobarbital (10 µM)	CYP2C19	< 20	-
Quinidine (1 µM)	CYP2D6	< 20	+
Ketoconazole (1 µM)	CYP3A4/5	82 ± 3	+ + +

+ + + > 50% inhibition of multiple metabolites, + + > 50% inhibition of 1 metabolite or > 20% inhibition of multiple metabolites, + > 20% inhibition of 1 metabolite,—< 20% inhibition of any metabolites

### In vitro inhibition and induction of cyps by entrectinib and m5

Studies in human liver microsomes demonstrated that entrectinib inhibited CYP3A4/5 with an IC_50_ of 2 μM (Table 2). The IC_50_ values for entrectinib and the other CYPs tested were > 10 μM. M5 appeared to be less inhibitory than entrectinib, with IC_50_ values of > 10 μM for all CYPs tested except for CYP2C8 (IC_50_ ~ 4.9 μM).

**Table 2:** In vitro inhibition of human drug metabolizing enzymes and transport proteins by entrectinib and M5

		IC_50_ (µM)	
		Entrectinib	M5
Drug Metabolizing Enzymes	CYP1A2	> 10	> 10
	CYP2B6	> 10	> 10
	CYP2C8	> 10	4.9
	CYP2C9	> 10	> 10
	CYP2C19	> 10	> 10
	CYP2D6	> 10	> 10
	CYP3A4/5	2.0	> 10
Transport Proteins	MDR1 (P-gp)	1.3	10

*CYP* cytochrome P450, *MDR* multidrug resistance, *P-gp* P-glycoprotein

Entrectinib was a very weak time-dependent inhibitor (TDI) of CYP3A4 but K_I_ and k_inact_ could not be measured in vitro as the TDI signal was too low

In vitro studies using human hepatocytes showed that entrectinib caused CYP3A4 mRNA induction by 48% of the positive control (rifampicin) at 10 μM and by 12.5% at 3 μM (Table S2). However, CYP3A enzyme activity was induced less than 10% of positive control using 10 μM entrectinib. M5 had no significant impact on the mRNA expression or activity of CYP3A4 (Table S3). Entrectinib did not significantly induce CYP1A2 mRNA or enzyme activity. Due to the positive CYP3A induction signal, induction of CYP2C enzymes was also investigated. Entrectinib induced CYP2C8 and CYP2C9 mRNA levels by 31% and 38%, respectively, of positive control levels at a test concentration of 3 μM, and by 90% and 109%, respectively, at a test concentration of 10 μM. M5 had no significant impact on the activities of the CYPs tested (CYPs 1A2, 2B6, 2C19, and 3A4).

### In vitro p-gp interactions with entrectinib and m5

Entrectinib showed a high efflux ratio in the in vitro P-gp assay, but did not display sensitivity to a P-gp inhibitor. In addition, entrectinib penetrated the brain in several in vivo models at steady-state, indicating a much lower P-gp effect at the level of the blood–brain barrier as suggested by the in vitro efflux ratio. Taken together, these data suggest entrectinib is a poor P-gp substrate. These data have recently been described by Fischer et al. [9] and will not be discussed further in the current manuscript. Entrectinib inhibits P-gp with an IC_50_ of 1.33 μM. M5 is both a substrate for and inhibitor of P-gp with an IC_50_ value of 10.1 μM.

### Subject disposition and demographics

In Study 1, a total of 20 subjects (10 per cohort) were enrolled and received treatment and 19 subjects completed the study. One subject in Cohort 1 withdrew during Period 2 prior to receiving entrectinib. Demographic characteristics were similar across both cohorts; all subjects were male, and the majority (75%) were White. Median age was 45 years (range 28 to 55 years) in Cohort 1, and 27.5 years (range 18 to 48 years) in Cohort 2.

In Study 2, a total of 15 subjects were enrolled, and 14 subjects received midazolam on Day 1 (ie, 1 subject was withdrawn by the investigator prior to treatment). Two subjects withdrew prior to receiving entrectinib; one subject withdrew due to progressive disease and 1 subject withdrew due to an adverse event. On Day 8, the remaining 12 subjects received midazolam 1 h after entrectinib. Of the 14 subjects who received treatment, the majority were female (64%), and White (93%) and the median age was 64.5 years (range 50 to 79 years).

In Study 3, 10 subjects were enrolled, received treatment, and completed the study. All subjects were male and the majority (80%) were White. Median age was 29.5 years (range 24 to 53 years).

### Pharmacokinetic results

#### Effect of a strong cyp3a4 inhibitor (Itraconazole) and strong cyp3a4 inducer (Rifampin) on the pharmacokinetics of single dose entrectinib (study 1)

**Fig. 1:**
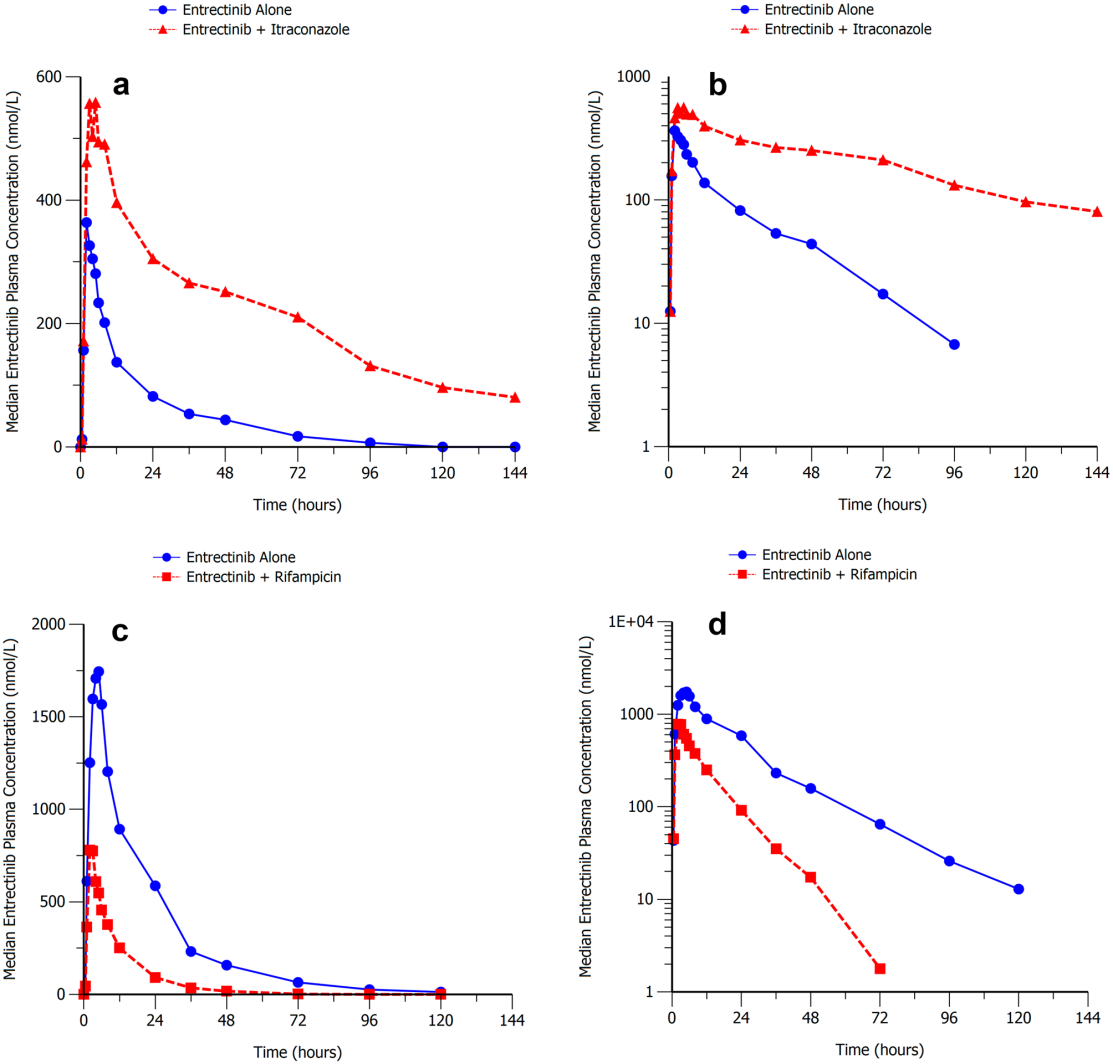
Median Entrectinib Plasma Concentration–Time Profiles Following a Single Oral Dose of Entrectinib Alone or With Multiple Dose Itraconazole (**a**, **b**, Upper Panels) or Rifampin (**c**, **d**, Lower Panels)

Median plasma concentration–time profiles for entrectinib in the presence and absence of itraconazole and rifampin are presented in Fig. 1. In Cohort 1, statistical analysis suggested that entrectinib C_max_, and AUC_inf_ were approximately 73%, and 504% higher, respectively when entrectinib was coadministered with itraconazole compared with entrectinib alone. Entrectinib median T_max_ occurred approximately 3 h later when entrectinib was administered with itraconazole compared with entrectinib alone (Table 3). The geometric mean t_1/2_ of entrectinib was notably longer when entrectinib was administered with itraconazole (50 h) compared with entrectinib alone (20 h). Entrectinib was partially converted to M5 with a median T_max_ of 5 and 6 h when entrectinib was administered alone and with itraconazole, respectively. Mean M5 t_1/2_ was notably longer when entrectinib was administered with itraconazole (88 h) compared with entrectinib alone (41 h). The mean M5/entrectinib ratios for AUC_inf_ showed that M5 exposure was approximately 29% of entrectinib exposure when entrectinib was administered alone, compared with 12% when it was coadministered with itraconazole.

**Table 3:** Summary of entrectinib and M5 plasma pharmacokinetic parameters with and without coadministration of itraconazole (200 mg QD) or rifampin (600 mg QD) following a single 100 or 600 mg entrectinib dose

Analyte	PK Parameter ^a^	Entrectinib 100 mg Alone (N = 10)	Entrectinib 100 mg with Itraconazole (N = 10)	Ratio of adjusted Geometric Means (90% CI)	Entrectinib 600 mg Alone (N = 10)	Entrectinib 600 mg with Rifampin (N = 10)	Ratio of Adjusted Geometric Means (90% CI)
Entrectinib	AUC_inf_ (nM•h)	6190 (50%)	36,100 (17%)	6.04 (4.54, 8.04)	36,300 (28%)	8440 (29%)	0.23 (0.18, 0.30)
	C_max_ (nM)	358 (35%)	615 (17%)	1.73 (1.37, 2.18)	1810 (25%)	807 (26%)	0.44 (0.35, 0.56)
	T_max_ (h)	2.0 (1.0, 3.0)	5.0 (2.0, 8.0)	ND	3.5 (2.0, 5.0)	2.0 (2.0, 5.0)	ND
	t_1/2_ (h)	20.2 (17%)	49.8 (22%)	ND	16.7 (16%)	9.9 (26%)	ND
M5	AUC_inf_ (nM h)	1710 (30%)	4310 (30%)	ND	11,000 (44%)	1530 (28%)	ND
	C_max_ (nM)	52.3 (37%)	31.5 (42%)	ND	383 (56%)	108 (23%)	ND
	T_max_ (h)	5.0 (3.0, 5.0)	6.0 (5.0, 48.0)	ND	5.0 (4.0, 5.0)	3.0 (2.0, 6.0)	ND
	t_1/2_ (h)	40.8 (22%)	88.0 (32%)	ND	33.8 (12%)	31.1 (28%)	ND
M5/Entrectinib ratio	AUC_inf_	0.288 (28%)	0.124 (29%)	ND	0.337 (56%)	0.187 (28%)	ND
	C_max_	0.151 (26%)	0.0538 (34%)	ND	0.234 (59%)	0.136 (23%)	ND

*AUC*_*inf*_ area under the plasma concentration–time curve extrapolated to infinity, *CI* confidence intervals, *C*_*max*_ maximum plasma concentration, *ND* not done, *t*_*1/2*_ terminal elimination half-life, *T*_*max*_ time of maximum plasma concentration

^a^Unadjusted geometric means (geometric coefficients of variation) for all except T_max_ which is median (minimum, maximum), and M5/entrectinib ratios which are arithmetic mean (coefficients of variation)

‘Unadjusted’ refers to raw data, and ‘adjusted’ refers to data that have been subjected to statistical analysis. Statistical comparisons were only conducted for entrectinib AUCinf and C_max_

In Cohort 2, statistical analysis suggested that entrectinib C_max_, and AUC_inf_ were approximately 56%, and 77% lower, respectively when entrectinib was coadministered with rifampin compared with entrectinib alone. Entrectinib median T_max_ occurred approximately 1.5 h earlier following administration of entrectinib with rifampin compared with entrectinib alone (Table 3). Entrectinib mean t_1/2_ was shorter (10 h) when administered with rifampin compared with entrectinib alone (17 h). The median T_max_ for M5 was 5 and 3 h for entrectinib alone and with rifampin, respectively. The mean t_1/2_ was estimated to be approximately 31 to 34 h following administration of entrectinib alone and with rifampin. The mean M5/entrectinib ratios for AUC_inf_ showed that M5 exposure was approximately 34% of entrectinib exposure when entrectinib was administered alone, compared with 19% of entrectinib exposure when coadministered with rifampin.

#### Effect of Entrectinib on the pharmacokinetics of cyp3a4 substrates (Midazolam and 1’-hydroxymidazolam; study 2)

**Fig. 2:**
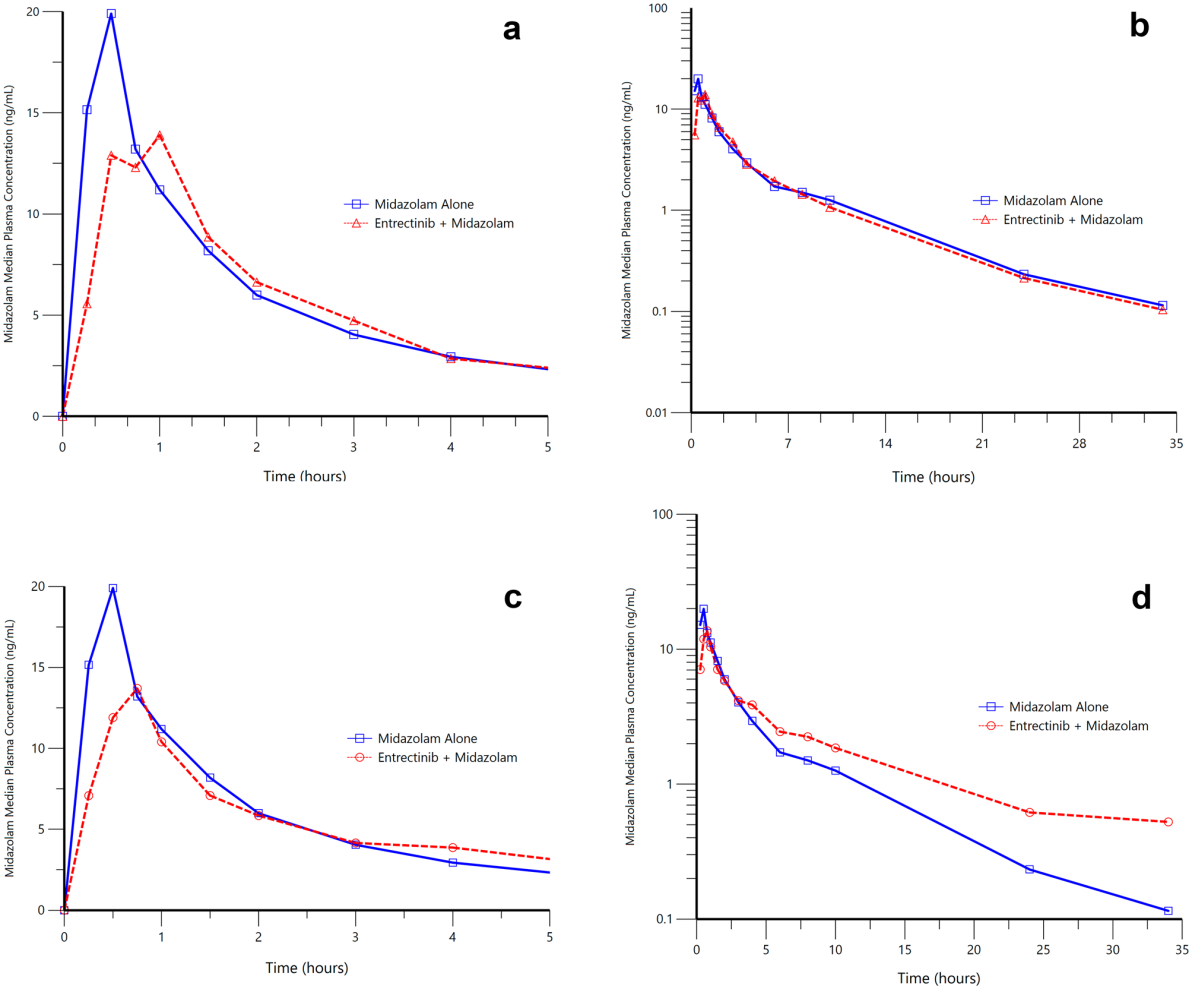
Median Midazolam Plasma Concentration–Time Profiles Following a Single Oral Dose of Midazolam Alone or With Single (**a**, **b**, Upper Panels) or Multiple (**c**, **d**, Lower Panels) Dose Entrectinib

Median plasma concentration–time profiles for midazolam in the presence and absence of entrectinib are presented in Fig. 2, and a summary of the PK parameters for midazolam and 1’-hydroxymidazolam are presented in Table 4. A single oral dose of entrectinib did not appear to affect the total exposure (AUC_inf_) of midazolam, with point estimates for the geometric mean ratios close to 1 and 90% CIs within 0.80 to 1.25. Peak midazolam concentrations (C_max_) were however reduced by approximately 35% following coadministration with single dose entrectinib. Multiple doses of entrectinib increased exposure of midazolam by approximately 50% (AUC_inf_), whereas C_max_ decreased by approximately 21%. Moderate inter-subject variability was observed for both midazolam and its metabolite with coefficients of variation (CV%) for C_max_ and AUC parameters of up to 60%. The mean t_1/2_ of midazolam was similar when midazolam was administered with a single dose of entrectinib (5.73 h) compared to alone (6.35 h), whereas administration of multiple doses of entrectinib with midazolam increased midazolam t_1/2_ to 8.13 h. Similar trends were observed for the AUC_inf_ and C_max_ of 1’-hydroxymidazolam when midazolam was administered in the presence and absence of single and multiple-dose entrectinib. The metabolic ratio of metabolite to parent for both AUC_inf_ and C_max_ was not affected by coadministration of single-dose entrectinib, but was decreased (point estimates of approximately 59% to 64%.) by coadministration with multiple-dose entrectinib.

**Table 4:** Summary of midazolam and 1’-hydroxymidazolam plasma pharmacokinetic parameters with and without coadministration of single and multiple dose entrectinib (600 mg/day) following a single 7.5 mg midazolam dose

Analyte	PK Parameter^a^	Midazolam Alone (N = 10)	Midazolam with Single Dose Entrectinib (N = 10)	Midazolam with Multiple Dose Entrectinib (N = 10)	Ratio of adjusted geometric means (90% Confidence Intervals)	
					Effect of Single Dose Entrectinib	Effect of Multiple Dose Entrectinib
Midazolam	AUC_inf_ (nM•h)	45.9 (61%)	43.7 (77%)	62.1 (79%)	1.00 (0.87, 1.16)	1.50 (1.29, 1.73)
	C_max_ (nM)	19.0 (64%)	12.9 (64%)	14.4 (50%	0.66 (0.56, 0.78)	0.79 (0.66, 0.94)
	T_max_ (h)	0.49 (0.25, 1.00)	0.75 (0.48, 23.6)^b^	0.50 (0, 1.05)	ND	ND
	t_1/2_ (h)	5.2 (68%)	5.33 (44%)	6.4 (68%)	ND	ND
1’-hydroxymidazolam	AUC_inf_ (nM•h)	14.9 (60%)	14.4 (52%)	14.0 (42%)	0.96 (0.83, 1.10)	0.89 (0.70, 1.12)
	C_max_ (nM)	6.57 (76%)	4.21 (48%)	3.51 (47%)	0.62 (0.50, 0.76)	0.49 (0.36, 0.67)
	T_max_ (h)	0.5 (0.25, 1.00)	1.0 (0.48, 23.6)	0.75 (0.5, 5.97)	ND	ND
	t_1/2_ (h)	3.71 (81%)	4.56 (49%)	5.37 (75%)	ND	ND

*AUC*_*inf*_ area under the plasma concentration–time curve extrapolated to infinity, *C*_*max*_ maximum plasma concentration, *ND* not done, *t*_*1/2*_ terminal elimination half-life, *T*_*max*_ time of maximum plasma concentration

^a^Unadjusted geometric means (geometric coefficients of variation) for all except T_max_ which is median (minimum, maximum)

^b^Maximum value was due to one outlying subject

Unadjusted’ refers to raw data, and ‘adjusted’ refers to data that have been subjected to statistical analysis. Statistical comparisons were only conducted for midazolam AUC_inf_ and C_ma_

#### Effect of entrectinib on the pharmacokinetics of a sensitive P-gp substrate (Digoxin; study 3)

**Fig. 3:**
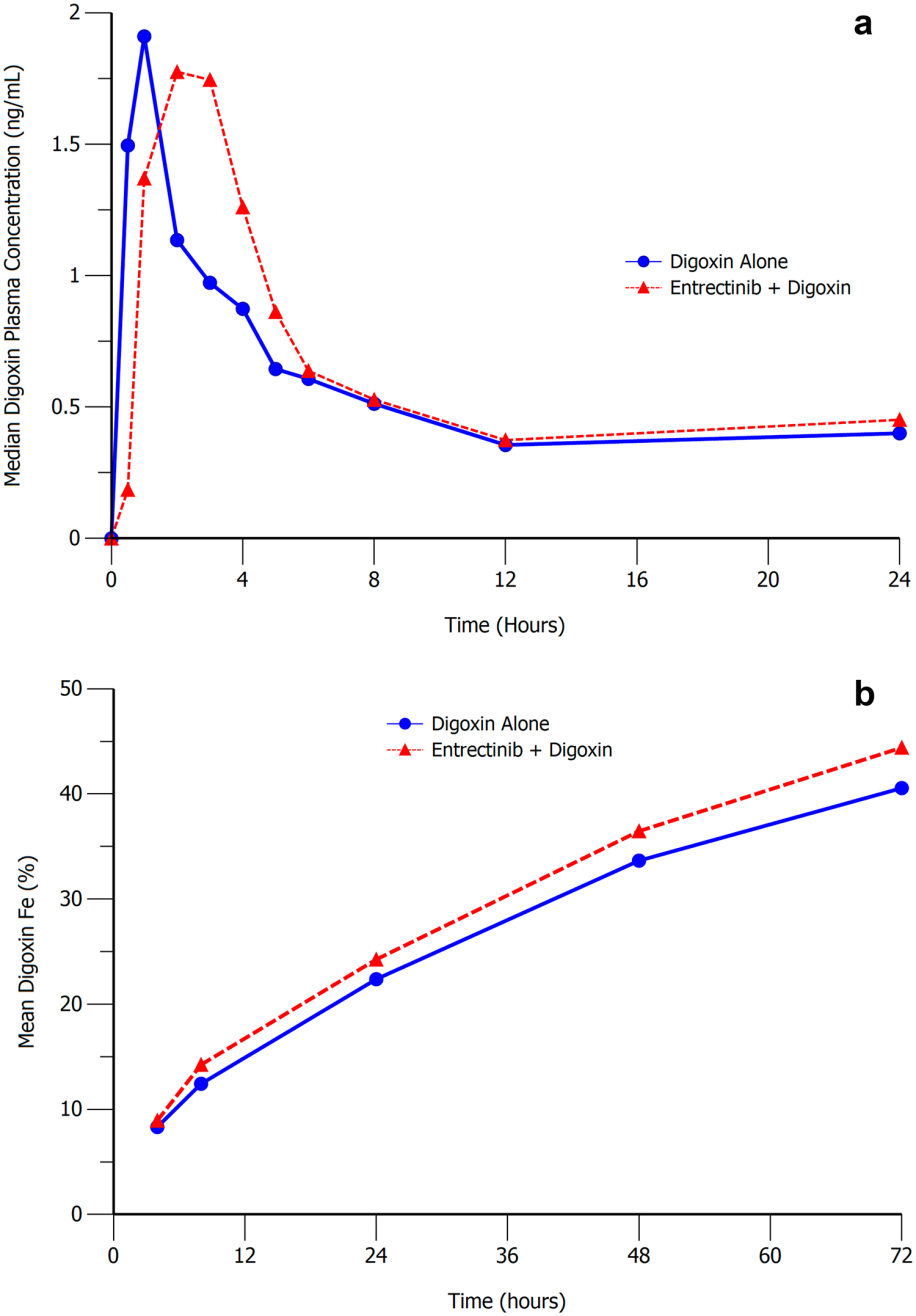
Median Digoxin Plasma Concentration–Time Profile (**a**, Upper Panel) and Mean Cumulative Urinary Excretion-time Profile (**b**, Lower Panel) Following a Single Oral Dose of Digoxin (0.5 mg) Alone or With a Single Oral Dose of Entrectinib (600 mg)

Median plasma concentration–time profiles and cumulative urinary excretion-time profiles for digoxin in the presence and absence of entrectinib are presented in Fig. 3. Coadministration of entrectinib with digoxin had a limited effect on the pharmacokinetics of digoxin (Table 5). Digoxin absorption was slower and T_max_ occurred approximately 1 h later when a single 600 mg dose of entrectinib was coadministered with digoxin. Digoxin peak (C_max_) and total exposure (AUC_inf_) after coadministration of entrectinib were 28% and 18% higher, respectively, than when digoxin was taken alone. The fraction of the digoxin dose excreted in urine was comparable (44% vs. 40%). Exploratory analysis showed that the geometric mean ratio of digoxin CL_R_ with and without coadministration of entrectinib was 94.6% (90% CI: 90.9%, 98.4%) indicating minimal effect of entrectinib on digoxin renal clearance.

**Table 5:** Summary of digoxin plasma pharmacokinetic parameters with and without coadministration of a single 600 mg dose of entrectinib following a single 75 mg digoxin dose

Analyte	PK parameter^a^	Digoxin Alone (N = 10)	Digoxin with Entrectinib (N = 10)	Ratio of adjusted geometric means (90% Confidence Intervals)
Digoxin	AUC_inf_ (nM•h)	34.2 (14%)	40.4 (17%)	1.18 (1.06, 1.32)
	C_max_ (nM)	1.97 (29%)	2.52 (39%)	1.28 (0.98, 1.67)
	T_max_ (h)	1.0 (0.5, 1.0)	2.0 (0.5, 3.0)	ND
	t_1/2_ (h)	35.9 (15%)	38.4 (37%)	ND
	CL_R_ (mL/min)	130 (16%)	123 (13%)	0.95 (0.91, 0.98)
	fe (%)	40.2 (13%)	44.0 (14%)	ND

*AUC*_*inf*_ area under the plasma concentration–time curve extrapolated to infinity, *CL*_*R*_ renal clearance, *C*_*max*_ maximum plasma concentration, *fe (%)* percent of dose excreted unchanged in urine, *ND* not done, *t*_*1/2*_ terminal elimination half-life, *T*_*max*_ time of maximum plasma concentration

^a^Unadjusted geometric means (geometric coefficients of variation) for all except T_max_ which is median (minimum, maximum)

‘Unadjusted’ refers to raw data, and ‘adjusted’ refers to data that have been subjected to statistical analysis. Statistical comparisons were only conducted for digoxin AUC_inf_, C_max_ and CL_R_

## Discussion

### Entrectinib as a victim of DDIs

Previous clinical studies with entrectinib have demonstrated that it is primarily cleared by metabolism and forms a major circulating and active metabolite (M5) that is thought to contribute to the clinical efficacy of the drug. Other, more minor pathways of metabolism included direct glucuronidation (M11) as well as N-oxidation and benzyl hydroxylation by CYP enzymes. Together these additional routes accounted for < 25% metabolism in vitro and none of the products were major circulating metabolites except M11 [7]. As M11 is a glucuronide conjugate which is not expected to show pharmacological activity, further in vivo examination of this metabolite was not required. The in vitro studies in human biomaterials described here showed that while several CYPs were able to metabolise entrectinib, the most important enzyme was CYP3A4. This enzyme was also shown to be involved in both the formation and the further metabolism of M5. To assess the relevance of these findings, a clinical study was conducted to investigate the magnitude of any changes in entrectinib exposure when entrectinib is coadministered with modulators of CYP3A4.

Coadministration of the strong CYP3A4 inhibitor itraconazole with entrectinib had a significant effect on entrectinib exposure with an increase in AUC_inf_ of approximately sixfold. M5 exposures also increased although to a lesser extent than the parent (AUC_inf_ increased by approximately 2.5-fold), with metabolite/parent ratios falling from approximately 29% to 12% in the presence of itraconazole. Coadministration of the strong CYP3A4 inducer rifampin with entrectinib also had a significant effect of entrectinib exposures with a decrease in AUC_inf_ of approximately 77%. M5 exposures also decreased although again to a lesser extent than the parent. The changes in M5 exposure when entrectinib was coadministered with itraconazole and rifampin confirm the in vitro finding that M5 is further metabolized by CYP3A4, and hence both its formation and clearance may be affected by modulators of CYP3A4 activity. Overall, these data show that entrectinib is a sensitive substrate of CYP3A4 with a fraction metabolized (f_m_) of 0.78 for entrectinib clearance estimated using physiologically-based pharmacokinetic (PBPK) modelling approaches (manuscript in preparation). Subsequent PBPK analysis has suggested that the dose of entrectinib should be reduced when coadministered with strong or moderate CYP3A4 inhibitors from 600 to 100 mg or 200 mg, respectively. Given the magnitude of reduction in entrectinib exposure when coadministered with rifampin, coadministration of entrectinib with strong CYP3A inducers is not recommended.

Itraconazole is also known to inhibit P-gp and in vitro studies suggested that entrectinib is a P-gp substrate, albeit a poor one. The limited effect of itraconazole on the peak concentrations of entrectinib after a single dose did not suggest that P-gp inhibition has a marked effect on entrectinib absorption. These data are consistent with the moderate to high entrectinib bioavailability implying that efflux transporters in gastrointestinal enterocytes do not significantly limit systemic drug availability.

### Entrectinib as a perpetrator of DDIs

The potential of entrectinib and M5 to inhibit or induce CYPs was also investigated in vitro. The studies in human liver microsomes suggested that entrectinib has the potential to inhibit CYP3A4/5 with an IC_50_ of 2 μM. The IC_50_ values for entrectinib with the other CYPs tested were > 10 μM, suggesting the potential for clinically relevant DDIs with these CYPs is low, and hence no formal clinical studies to assess possible interactions with substrates of these CYPs has been conducted. M5 had IC_50_ values > 10 μM for several CYPs except for CYP2C8 where the IC_50_ was approximately 4.9 μM. The estimated free C_max_ of M5 in plasma from patients treated at 600 mg QD is 0.0134 μM, which is > 300-fold lower than the IC_50_, and hence the potential for entrectinib to cause DDIs with CYP2C8 substrates via M5 is considered to be low. Note, the actual unbound fraction of M5 is < 1% in human plasma (Roche data on file), but a value of 1% has been used for initial risk assessment based on FDA guidance [10].

Given the potential for entrectinib to inhibit and/or induce CYP3A4, a clinical study was conducted to determine the effect of entrectinib on midazolam, which is a sensitive index CYP3A4 substrate recommended for such assessments [11]. Baseline (Day 1) midazolam and 1’-hydroxymidazolam plasma concentrations and PK parameters were generally consistent with literature reports [12, 13]. Coadministration of midazolam with a single dose of entrectinib did not have a significant effect on midazolam total exposure (AUC_inf_) although peak plasma concentrations were reduced by approximately 35%. It should be noted that the observed change in C_max_ is not anticipated to be due to enzyme induction, but rather may be due to decreased rate or extent of midazolam absorption when administered in combination with entrectinib. For example, midazolam exposure has previously been shown to be susceptible to food and changes in gastric pH [14, 15], and therefore its absorption may be affected by the acidifying agent included in the entrectinib formulation (to aid entrectinib’s absorption and reduce variability)[7, 16]. The metabolic ratios of midazolam to its metabolite for both AUC_inf_ and C_max_ were not affected by single dose entrectinib.

Multiple oral doses of entrectinib at steady-state increased midazolam exposure by approximately 50% although as with single dose entrectinib, midazolam C_max_ decreased (approximatley 20%). However, 1’-hydroxymidazolam total exposure was not changed, and the 1’-hydroxymidazolam metabolite-to-parent ratio was decreased by 41%. These data are consistent with inhibition of 1’-hydroxymidazolam formation.

Overall, the data from the midazolam study suggest that repeat dosing with entrectinib had limited influence on midazolam total exposure indicating that entrectinib is a weak inhibitor of CYP3A4. No dose adjustment is recommended when entrectinib is coadministered with CYP3A4 substrates.

In vitro studies suggested that entrectinib may also induce CYP2C8 and CYP2C9 although to a lesser extent than CYP3A4. The lack of induction of CYP3A activities observed in the clinical midazolam study indicates that induction of CYP2C enzyme activities is unlikely, as both enzymes share a common mechanism of regulation (ie, via the pregnane xenobiotic receptor [PXR])[17]. It has been noted that PXR agonists, such as rifampin, rifabutin and carbamezipine, cause dose-dependent interactions with CYP2C9 and P-gp that are one category lower (eg, strong to moderate or moderate to mild) than those observed for CYP3A4[18, 19]. Therefore, as entrectinib did not induce CYP3A4 in clinical studies despite the potential observed in vitro, no induction of CYP2C9 or P-pg was expected. Clinical studies of CYP2C enzyme induction were therefore not performed.

As entrectinib and M5 have the potential to inhibit P-gp, a clinical study with digoxin was conducted. Digoxin is commonly used as a probe P-gp substrate given the results are relevant due to its narrow therapeutic window[11]. A single oral dose of entrectinib coadministered with digoxin was shown to increase digoxin exposure by 20–30% indicating that entrectinib is a weak P-gp inhibitor in the gut. No significant effect was noted on digoxin renal clearance, suggesting a minor role of renal P-gp inhibition by entrectinib. Given the magnitude of the effects observed, no dose adjustment is required when entrectinib is coadministered with P-gp substrates.

In summary, entrectinib is a sensitive CYP3A4 substrate that is subject to clinically relevant DDIs when coadministered with moderate/strong CYP3A4 inhibitors and strong CYP3A4 inducers. A dose reduction from 600 mg QD to 200 mg QD and 100 mg QD, is recommended when entrectinib is coadministered with moderate and strong CYP3A4 inhibitors, respectively [20]. Entrectinib is not recommended to be administered with moderate or strong CYP3A4 inducers. Entrectinib is also a weak inhibitor of CYP3A4 although no clinically significant interaction with a sensitive CYP3A4 substrate was observed. Entrectinib does not induce CYP3A4 in vivo at the recommended dose and therefore clinically relevant interactions with CYP3A4 (or CYP2C) substrates are not expected. No clinically relevant interaction was observed with a P-gp substrate and therefore entrectinib can be coadministered with P-gp substrates without dose adjustment.

## Supplementary information

Below is the link to the electronic supplementary material.

Supplementary file1 (DOCX 25 KB)

## Data Availability

Qualified researchers may request access to individual patient level data through the clinical study data request platform (https://vivli.org/). Further details on Roche's criteria for eligible studies are available here (https://vivli.org/members/ourmembers/). For further details on Roche's Global Policy on the Sharing of Clinical Information and how to request access to related clinical study documents, see here (https://www.roche.com/research_and_development/who_we_are_how_we_work/clinical_trials/our_commitment_to_data_sharing.htm).
