# Supplementary material for: In vitro and clinical investigations to determine the drug-drug interaction potential of entrectinib, a small molecule inhibitor of neurotrophic tyrosine receptor kinase (NTRK)
Source: Invest New Drugs. 2021 Aug 21;40(1):68–80. doi: 10.1007/s10637-021-01156-9 (PMC8763936; doi:10.1007/s10637-021-01156-9)
Supplement: Supplementary file 1 — Supplementary file1 (DOCX 25 KB) [file 10637_2021_1156_MOESM1_ESM.docx]

**SUPPLEMENTAL MATERIAL**

***In Vitro and Clinical Investigations to Determine the Drug-Drug Interaction Potential of Entrectinib***

*Investigational New Drugs*

Georgina Meneses-Lorente*; Stephen Fowler; Elena Guerini; Karey Kowalski; Edna Chow-Maneval; Li Yu; Francois Mercier; Mohammed Ullah; Kenichi Umehara; Andreas Brink; Vincent Buchheit; Elke Zwanziger; Alex Phipps; Nassim Djebli

*Georgina Meneses-Lorente, Roche Products Ltd, Welwyn Garden City, UK;
Email: [georgina.meneses-lorente@roche.com](mailto:georgina.meneses-lorente@roche.com)

**In Vitro Metabolism Methods**

Experiments were performed using standard methodologies. Key details of the study designs are listed below.

**Effect of CYP-Selective Chemical Inhibitors on Metabolism of Entrectinib by Human Liver Microsomes**

| Test substrates | Entrectinib (1 μM )  Control Cocktail 1: 1 μM Tacrine, 10 μM Bupropion, 1 μM Dextromethorphan  Control Cocktail 2: 1 μM Diclofenac, 1 μM Midazolam, 1 μM Amodiaquine  Control Cocktail 3: 25 μM *S*-Mephenytoin |
| --- | --- |
| Test Inhibitors | 0.5μM α-Naphthoflavone, 50μM Ticlopidine, 3μM Montelukast, 10μM Sulfaphenazole, 2μM Benzylphenobarbital, 1μM Quinidine, 1μM Ketoconazole |
| Incubation system | Human liver microsomes (150-donor mixed gender pool, 1 mg/mL final concentration)  100 mM Phosphate buffer (pH7.4)  NADPH (1 mM) |
| Experimental Method | Incubations warmed to 37°C over 5 mins.  Incubations started by addition of cofactor. 40 μL aliquots of each incubation removed at 0.5, 3.5, 6.5, 10, 15, 20, 25 & 30 minutes and added to 80 μL acetonitrile containing D6-midazolam as internal standard.  Quenched samples cooled for 1 hour then centrifuged, supernatant removed and 1µL of each sample was analysed by LC-MS/MS using a Q-exactive Orbitrap mass spectrometer for entrectinib samples and an AB Sciex API6500 triple quadrupole mass spectrometer for positive control substrates.  Drug substances and metabolites analyzed. Peak area ratios vs internal standard used in data analysis. Effect of the inhibitory compounds on the intrinsic clearance of amodiaquine, diclofenac and midazolam was assessed. Effect of initial metabolite formation rates for selective metabolism reactions of the other control compounds was assessed. |

**Entrectinib Metabolism by Individual CYP and FMO Enzymes**

| Test substrates | Entrectinib (10 μM )  Phenacetin (CYP1A1), Tacrine (CYP1A2), Coumarin (CYP2A6), Bupropion (CYP2B6), Paclitaxel (CYP2C8), Diclofenac (CYP2C9 and CYP2C18), S-Mepheytoin (CYP2C19), Dextromethorphan (CYP2D6), Chlorzoxazone (CYP2E1), Midazolam (CYP3A4 and CYP3A5) and Benzydamine (FMOl and FM03). All incubations used 1µM positive control substrate with the exception of 25µM diclofenac in the CYP2C18 incubation. |
| --- | --- |
| Incubation systems | Recombinantly expressed individual enzymes sourced from Corning-Gentest at concentrations of 20 pmol/mL (with 40 pmol/mL for CYP2C18 and CYP2E1)  human liver microsomes (50-donor mixed gender pool, Lot LBB, Celcis)  Dulbecco’s buffer, pH 7.4,  NADPH (1 mM) |
| Incubation Times (min) | 10, 30, 60 minutes |
| Experimental Method | Metabolism was started by the addition of NADPH (final concentration 1 mM) after 5 min pre-incubation time. Incubations were performed in 96 well plates, under shaking. At t=0, and after 10, 30 and 60 minutes, aliquots of the incubate solution were taken. Metabolism was be stopped by the addition of acetonitrile containing (injection check); samples were centrifuged and the supernatant was analyzed by LC-MS/MS to investigate the disappearance of compound. |

**Direct and time-dependent inhibition of major CYP isoforms by entrectinib**

| Test substrates and incubation times | Phenacetin (CYP1A2, 31µM, 20 mins, 0.125mg/ml HLM), Efavirenz (CYP2B6, 5µM, 30mins, 0.125mg/mL HLM), Amodiaquine (CYP2C8, 1µM, 5mins, 0.125mg/mL HLM), Diclofenac (CYP2C9, 5µM, 10 mins, 0.125mg/mL HLM), S-Mepheytoin (CYP2C19, 22.5µM, 30mins, 0.25mg/mL HLM), R-bufuralol (CYP2D6, 12.5µM, 15mins, 0.1 mg/mL HLM), Midazolam (CYP3A4/5, 3µM, 2 mins, 0.1mg/ml HLM) and Testosterone (CYP3A4/5, 16 µM, 10 mins, 0.05 mg/ml HLM) |
| --- | --- |
| Test Inhibitors | Reversible inhibitors: alpha-Naphthoflavone (CYP1A2), tamoxifen (CYP2B6), quercetin (CYP2C8), sulphaphenazole (CYP2C9), N-3-benzylnirvanol (CYP2C19), quinidine (CYP2D6), ketoconazole (CYP3A4/5).  Time-dependent inhibitors: furafylline 8CYP1A2), thio-TEPA (CYP2B6), gemfibrozil glucuronide (CYP2C8), tienilic acid (CYP2C9), S-fluoxetine (CYP2C19), paroxetine (CYP2D6), troleandomycin (CYP3A4/5) |
| Incubation systems | Human liver microsomes: Mixed gender pool of 200 donors, Lot 1410230, Xenotech. |
| Experimental Method | Reversible inhibition: Inhibitor (1 μL) was added to 88 μL of HLM in 100 mM sodium phosphate buffer, pH 7.4, followed by addition of the probe substrate (1 μL) to the incubation mixture. The incubation plate was pre-incubated for 3 minutes at 37°C. 10 mM NADPH (10 μL) was added to initiate the activity reaction for a final concentration of 1 mM. The samples were incubated for the appropriate incubation time for probe substrate reaction.  Time- plus NADPH-dependent inhibition: Inhibitor (1 μL) was added to 88 μL of HLM in 100 mM sodium phosphate buffer, pH 7.4, followed by addition of 10 μL of 10 mM NADPH (final concentration of 1 mM). The plate was pre-incubated for 30 minutes at 37°C. The probe substrate (1 μL) was added to the incubation mixture to initiate the activity reaction. The samples were incubated for the appropriate incubation time for the probe substrate reaction. |

**Induction of CYP isoforms by entrectinib and M5**

| Test substrates and incubation times | RT-PCR analysis of CYP1A2, CYP2C8, CYP2C9, CYP2C19, CYP3A4 mRNA normalized using GAPDH  Phenacetin (CYP1A2, 100µM, 45mins), S-Mephenytoin (CYP2C19, 400µM, 30mins), Midazolam (CYP3A4/5, 30µM, 45mins) |
| --- | --- |
| Test Inducers | Omeprazole (50µM), Phenobarbital (750µM), Rifampin (20µM), Flumazenail (-ve control, 25µM), DMSO (vehicle control), Entrectinib (1-100µM), Entrectinib-M5 (0.1-50µM) |
| Incubation systems | Cryopreserved individual donor human hepatocytes, XenoTech |
| Experimental Method | Plated individual donor human hepatocytes were incubated at 37°C in a 5% CO_2_ 95% humidity incubator, with the test inducers for 3 days with daily replacement of media/inducing agents. After the 3^rd^ day RNA preparation was made from some wells whilst others were incubated with fresh media containing CYP substrates. |

**Table S1: Qualitative overview of entrectinib metabolism by recombinantly expressed CYP Enzymes**

| Recombinantly Expressed Enzyme | Entrectinib Metabolism to M5 | Entrectinib Metabolism  to other Products | M5 Metabolism |
| --- | --- | --- | --- |
| CYP1A1 | ++ | + | + |
| CYP1A2 | - | - | - |
| CYP2B6 | - | - | - |
| CYP2C8 | + | + | - |
| CYP2C9 | - | - | - |
| CYP2C19 | ++ | - | - |
| CYP2D6 | + | - | - |
| CYP3A4 | +++ | +++ | +++ |
| CYP3A5 | ++ | + | ++ |
|  |  |  |  |

+++ high turnover, ++ moderate turnover, + low turnover, - little or no turnover. NA: not applicable; ND: not determined.

**Table S2: Induction of major CYP enzymes by entrectinib in human hepatocytes**

| **CYP Enzyme** | **Mean ± SD Fold Change Based on CYP mRNA** | | **Relative Effectiveness ± SD Based on CYP mRNA** | | **Relative Effectiveness  Based on CYP Activity** | |
| --- | --- | --- | --- | --- | --- | --- |
|  | **3 μM** | **10 μM** | **3 μM** | **10 μM** | **3 μM** | **10 μM** |
| CYP3A4 | 2.63 ±1.07 | 7.22 ±3.61 | 12.5% ±11.0 | 47.9% ±38.3 | No significant change | No significant change |
| CYP2C8 | 2.08 ±0.62 | 4.24 ±1.48 | 30.8% ±16.2 | 90.2% ±28.6 | ND | ND |
| CYP2C9 | 1.87 ±0.29 | 3.46 ±0.62 | 38.3% ±7.7 | 109% ±9 | ND | ND |
| CYP2C19 | ND | ND | ND | ND | No significant change | No significant change ^a^ |
| CYP2B6 | ND | ND | ND | ND | ND | ND |
| CYP1A2 | No significant change | No significant change | No significant change | No significant change | No significant change | No significant change |

CYP = cytochrome P450; ND = not determined.

Values represent the average of three individual cultures for the specified analysis endpoint unless otherwise noted.

Relative effectiveness (percent positive control, %) = (Fold change of test article treated cells – fold change of vehicle control) / (Fold change of positive control – fold change of vehicle control) x 100

a n=2

**Table S3: Induction of major CYP enzymes by M5 in human hepatocytes**

| **CYP Enzyme** | **Relative Effectiveness ± SD Based on CYP mRNA** | | | **Relative Effectiveness ± SD Based on CYP Activity** | | |
| --- | --- | --- | --- | --- | --- | --- |
|  | **0.5 μM** | **2.5 μM** | **10 μM** | **0.5 μM** | **2.5 μM** | **10 μM** |
| CYP3A4 | 1.16% ±1.47 | 5.9% ±5.01 | 16.5% ±11.2 | 1.25% ±1.17 | 4.61% ±3.06 | 5.42% ±4.45 |
| CYP2C19 | ND | ND | ND | −1.21% ±0.94 | −3.22% ±3.41 | −9.87% ^a^ |
| CYP2B6 | 4.22% ±4.37 | 19.3% ±11.4 | 26.5% ±10.7 | 2.98% ±2.91 | 9.02% ±8.75 | 9.56% ±8.52 |
| CYP1A2 | 0.22% ±0.23 | 1.22% ±0.87 | 1.70% ±0.15 | 0.575% ±0.657 | 1.30% ±1.4 | 0.445% ±0.27 |

CYP = cytochrome P450; ND = not determined.

Values represent the average of three individual cultures for the specified analysis endpoint unless otherwise noted.

Relative effectiveness (percent positive control, %) = (Fold change of test article treated cells – fold change of vehicle control) / (Fold change of positive control – fold change of vehicle control) x 100

a n=2
